# Therapeutic effects of electrical stimulation on overactive bladder: a meta-analysis

**DOI:** 10.1186/s40064-016-3737-5

**Published:** 2016-11-29

**Authors:** De Ting Zhu, Xiao Jun Feng, Yun Zhou, Jian Xian Wu

**Affiliations:** Department of Rehabilitation Medicine, the Second Hospital of Anhui Medical University, Hefei, 230601 China

**Keywords:** Electrical stimulation, Overactive bladder, Meta-analysis, Randomized controlled trials

## Abstract

**Background:**

To systematically evaluate the therapeutic effect of electrical stimulation (ES) on overactive bladder (OB).

**Method:**

We retrieved information by searching databases from PubMed, CBM-disc, The Cochrane Library, ScienceDirect (from Elsevier publishers) and Springer publishers up to March 2016. We looked for randomized controlled trials that studied ES in OB treatment with subject headings and keywords using literature searches and manual retrieval. References of included studies were reviewed. Literature was screened independently by two investigators according to inclusion and exclusion criteria. After extracting data and evaluating their quality, meta-analysis was undertaken with RevMan v5.2.

**Results:**

Ten randomized controlled trials involving 719 patients were included. Meta-analysis results demonstrated ES to have better effects for improving bladder compliance, reducing residual urine, and decreasing the frequency of enuresis in OB patients compared with the control group. ES elicited significantly better effects for diminishing the maximum detrusor pressure in children than in controls, but there was no significant difference in the maximum detrusor pressure between adults and controls. The therapeutic effect of ES combined with other therapies for increasing the maximum bladder capacity was better compared with other therapies alone. No significant difference was noted between ES alone and other therapies alone.

**Conclusions:**

Based on current evidence, ES has certain effects on OBs. Severe adverse reactions are not observed. ES is safe, efficacious, and worthy of clinical use.

## Background

Urgency is the main clinical manifestation of overactive bladder (OB). Often, OB is accompanied by frequent urination and enuresis, and may be associated with/without urge incontinence. OB can be expressed as overactivity of detrusor muscle or other forms of urethra–bladder dysfunction (Abrams et al. 2002). Excessive activity of the bladder can result if uncontrolled suppression and abnormal excitement occur in the central nervous system, peripheral nervous system, or smooth muscle of the bladder. Often, OB is accompanied by dysfunction of the bladder and urethra. Such dysfunction can induce an infection in the urinary tract, stones or ureteral reflux which, if severe, may result in death due to renal failure. Often, urgency, urinary frequency and urinary incontinence reduce the ability to work and to participate in social activities, and can result in a poor social functioning. Therefore, restoration of urinary continence is important for improving the quality and prolongation of the lives of OB patients.

Conventional treatments for OB are drug therapy, bladder rehabilitation training, and surgery. Drug treatment is based mainly on anticholinergic agents. The efficacy of these drugs is good, but often the patient cannot adhere to medication because of adverse reactions (Pelletier et al. 2009). Bladder rehabilitation training can take a long time, and often the effect is not satisfactory. Surgery is traumatic, is associated with complications, and is not accepted by some patients.

In recent decades, electrical stimulation (ES) has been developed and used for treatment of dysfunction of the lower urinary tract. Several studies have shown ES to be effective for treating dysfunction of the lower urinary tract by increasing the capacity or compliance of the bladder or possibly by decreasing detrusor pressure (Bosch and Groen 1998; Amaro et al. 2006; Lee et al. 2012a).

However, some questions regarding ES need to be answered: can ES improve bladder function-related indicators? What is the degree of improvement? Are there differences in therapeutic effects comparable with conventional treatment? What are the adverse reactions of ES? Is ES safe? Can ES be an alternative to conventional treatment?

This study sought to: (1) summarize the findings of published randomized controlled trials (RCTs); (2) comprehensively and objectively understand the therapeutic effect of ES for bladder dysfunction; (3) provide effective guidance for clinical treatment.

## Methods

### Study design

The present study was based on RCTs regardless of whether a blinding method was used.

### Study subjects

Urgency is the main clinical manifestation of OB. Often, OB is accompanied by frequent urination and enuresis, and may be with or without urge incontinence. OB can be expressed as overactivity of detrusor muscle or other forms of urethra–bladder dysfunction.

### Interventions

The trial group underwent ES or ES combined with other therapies irrespective of stimulation mode, parameter setting, or treatment course. The control group underwent other therapies (except ES), such as anticholinergic drugs and ES without an electric current.

### Exclusion criteria

There were six exclusion criteria: (1) duplicate detection or duplicate publication; (2) non-RCTs; (3) case–control study; (4) Review-, Discussion- or Comment-type articles; (5) raw data could not be used; (6) useful data could not be obtained by contacting the corresponding author.

### Outcome measures

Outcome measures were: (1) maximum bladder capacity; (2) maximum detrusor pressure; (3) bladder compliance; (4) residual urine; (5) enuresis.

### Retrieval strategy

We retrieved information by searching databases from PubMed, CBM-disc, The Cochrane Library, ScienceDirect (from Elsevier publishers) and Springer publishers up to March 2016. We looked for RCTs that studied ES in the treatment of OB with subject headings and keywords using literature searches and manual retrieval. Search terms were: “nerve stimulation”, “electrical stimulation”, “neuromodulation”, “electroacupuncture”, “incontinence”, “lower urinary tract dysfunction”, “neurogenic bladder”, “urinary retention”, “multiple sclerosis”, “spinal cord injury”, “cerebral vascular disease”, “Parkinson’s disease”, “Alzheimer’s disease”, “detrusor hyperreflexia”, “detrusor overactivity”, and “detrusor sphincter dyssynergia”.

### Document screening and data extraction

Literature was screened independently by two investigators according to the inclusion and exclusion criteria described above. Information was collected with a pre-designed data-extraction form that included: (1) basic information (author, publication year, baseline data); (2) trial design, interventions, measurement index and duration of follow-up. Disagreements were resolved by discussion or through consensus with a third investigator. Incomplete information was supplemented by contacting the corresponding author of the article. If we could not obtain original data, the study was excluded.

### Assessment of methodological quality of included studies

The risk of bias of the included RCTs was evaluated independently with the bias risk-assessment tool in the *Cochrane Handbook for Systematic Reviews of Interventions* (v5.1.0) by two researchers. Contents include whether: (1) the correct method of randomization has been adopted; (2) allocation concealment has been used; (3) intervention measures have been implemented by a blinded method; (4) outcome indicators have been evaluated by a blinded method; (5) all results have been reported (number of patients lost to follow-up, number of dropouts, intent-to-treat analysis); (6) there has been selective reporting of results; (7) there are other sources of bias. Disagreements were resolved by discussion or through consensus with a third investigator.

### Statistical analyses

Data were analyzed using RevMan v5.2. Measurement data used the mean difference (MD) or standardized mean difference (SMD) and its 95% confidence interval (CI). Count data used odds ratios (ORs) and 95% CI for efficacy analyses. Heterogeneity testing among results was conducted with the χ^2^ test. For statistical homogeneity (*P* > 0.1, *I* ^2^ < 50%), a meta-analysis was done using fixed-effect models. For statistical heterogeneity (*P* < 0.1, *I* ^2^ > 50%), the source of heterogeneity was analyzed, and a subgroup analysis of the factors that might lead to heterogeneity was carried out. If there was statistical heterogeneity among study results but clinical heterogeneity was absent, a meta-analysis was undertaken using random-effect models. If the heterogeneity between the two groups was too large or we could not find the data source, descriptive analysis was used. For all observed indicators, the level of significance was set at alpha = 0.05.

## Results

### Results of literature retrieval

**Fig. 1:**
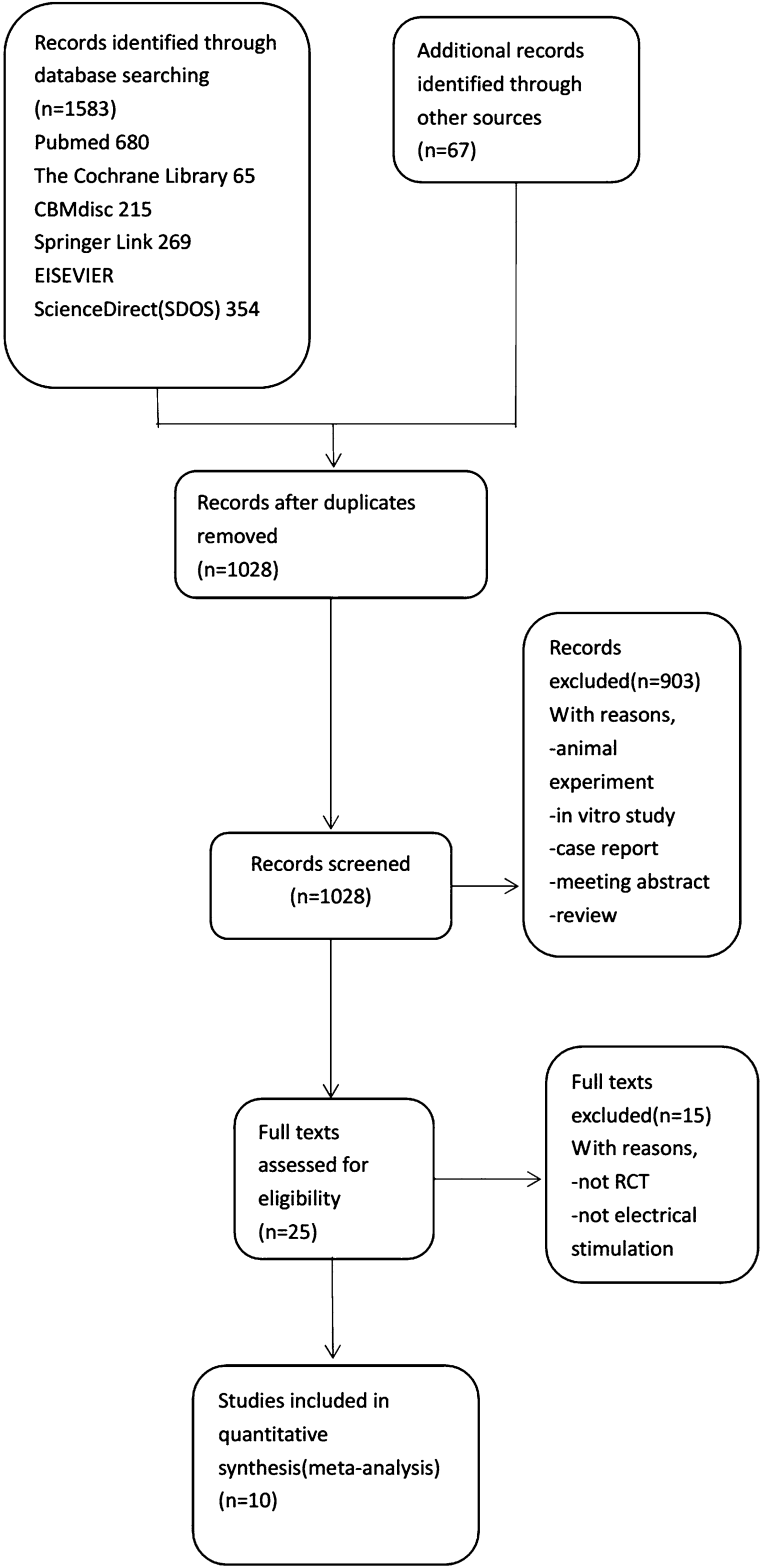
Flowchart and results of literature screening

A total of 1583 studies were screened primarily. 1558 studies were excluded by eliminating duplication, and reading the title and abstract. Finally, 10 RCTs involving 719 patients were included by reading the full-text. A flowchart and results of literature screening are displayed in Fig. 1. Basic characteristics of each study are listed in Table 1 (Chen et al. 2015; McClurg et al. 2008; Kajbafzadeh et al. 2009, 2014; Meng et al. 2015; Yamanishi et al. 2000; Chen et al. 2012; Peters et al. 2009, 2010; Arruda et al. 2008).

**Table 1:** Basic characteristics of included studies

Source	Chen et al. (2015)	McClurg et al. (2008)	Kajbafzadeh et al. (2014)	Kajbafzadeh et al. (2009)	Meng et al. (2015)	Yamanishi et al. (2000)	Chen et al. (2012)	Peters et al. (2009)	Peters et al. (2010)	Arruda et al. (2008)
No. of patients (T/C)	50/48	37/37	15/15	19/10	20/15	32/26	30/30	41/43	103/105	21/22
No. of female (%) (T/C)	6/6	84/70	46/34	63/60	Unknown	62/52	37/43	96/92	78.2/80	100/100
Mean age, year (T/C)	32.9 ± 1.8/ 33.5 ± 1.7	48.3 ± 11.5/ 52.0 ± 8.8	5.9 ± 3.5/ 7.4 ± 3.6	5.7 ± 2.8/ 5.6 ± 2.4	36 ± 8/ 36 ± 7	67.5 ± 12.7/ 73.0 ± 8.7	65.60 ± 3.79/ 61.93 ± 3.67	57.5 ± 15.2/ 58.2 ± 11.3	62.5/ 60.2	35–80
Duration of disease, years (T/C)	3.5 ± 1.4/ 3.3 ± 1.7	Unknown	Unknown	Unknown	1.2 ± 0.7	4.7 ± 5.2/ 4.6 ± 8.0	5.4 ± 1.75	9.8 ± 12.3/ 9.4 ± 12.1	10.2 ± 11.5/ 9.8 ± 10.4	Unknown
Cause of OAB	SCI	MS	Myelomeningocele	Myelomeningocele	SCI	Unknown	PD	Unknown	Unknown	Unknown
Design	RCT	RCT	RCT	RCT	RCT	RCT	RCT	RCT	RCT	RCT
Intervention	T:PTNS C:solifenacin succinate (5 mg/d)	T:PFMT + EMG biofeedbock + active NMES C:PFMT + EMG biofeedbock + placebo NMES	T:FES C:sham stimulation	T:IFS C: sham stimulation	T:Electroacupuncture +BTX-A + Rehabilitation training C:BTX-A + Rehabilitation training	T:DP/CNS C:sham stimulation	T:Electroacupuncture + Tolterodine(1 mg twice a day) C:tolterodine(2 mg twice a day)	T:PTNS C:tolterodine(4 mg/d)	T:PTNS C:sham stimulation	T:FES C:PFMT
Duration of the treatment	4 weeks	9 weeks	15 courses	2 weeks	4 weeks	4 weeks	6 weeks	12 weeks	12 weeks	12 weeks
Outcome Measures	①	①④	①②③⑤	①②③④⑤	①②④	①②③	①	①⑤	①⑤	①②⑤
Follow-up,weeks	16.24	6	79 ± 5	38						

*OAB* overactive bladder, *SCI* spinal cord injury, *MS* multiple sclerosis, *PD* Parkinson’s disease, *PTNS* percutaneous tibial nerve stimulation, *PFMT* pelvic floor muscle training, *EMG* electromyography, *NMES* neuromuscular electrical stimulation, *FES* functional electrical stimulation, *IFS* interferential electrostimulation, *DP/CNS* dorsal penile/clitoral nerve stimulation

Outcome measures: ① Mean maximum bladder capacity; ② Mean maximum detrusor pressure; ③ Mean bladder compliance; ④ Residual urine; ⑤ Enuresis

The 10 studies compared baseline data between the trial group and control group. Of these studies, six studies reported the duration of disease (the shortest was 1 year, and the longest was 10 years). Six studies reported the reasons for OB. Injury to the spinal cord was noted in two studies, spinal myelomeningocele in two studies, multiple sclerosis in one study, and Parkinson’s disease in one study. The shortest duration of ES was 2 weeks and the longest was 12 weeks. Among seven studies, the difference was only in the effect of ES and, in the other three studies, the difference was between ES and other treatments. The ES mode was percutaneous stimulation of the tibial nerve in three studies, neuromuscular ES in one study, functional ES in two studies, interferential electrostimulation in one study, electroacupuncture in two studies, and dorsal penile/clitoral nerve stimulation in one study.

### Assessment of the methodological quality of included studies

**Fig. 2:**
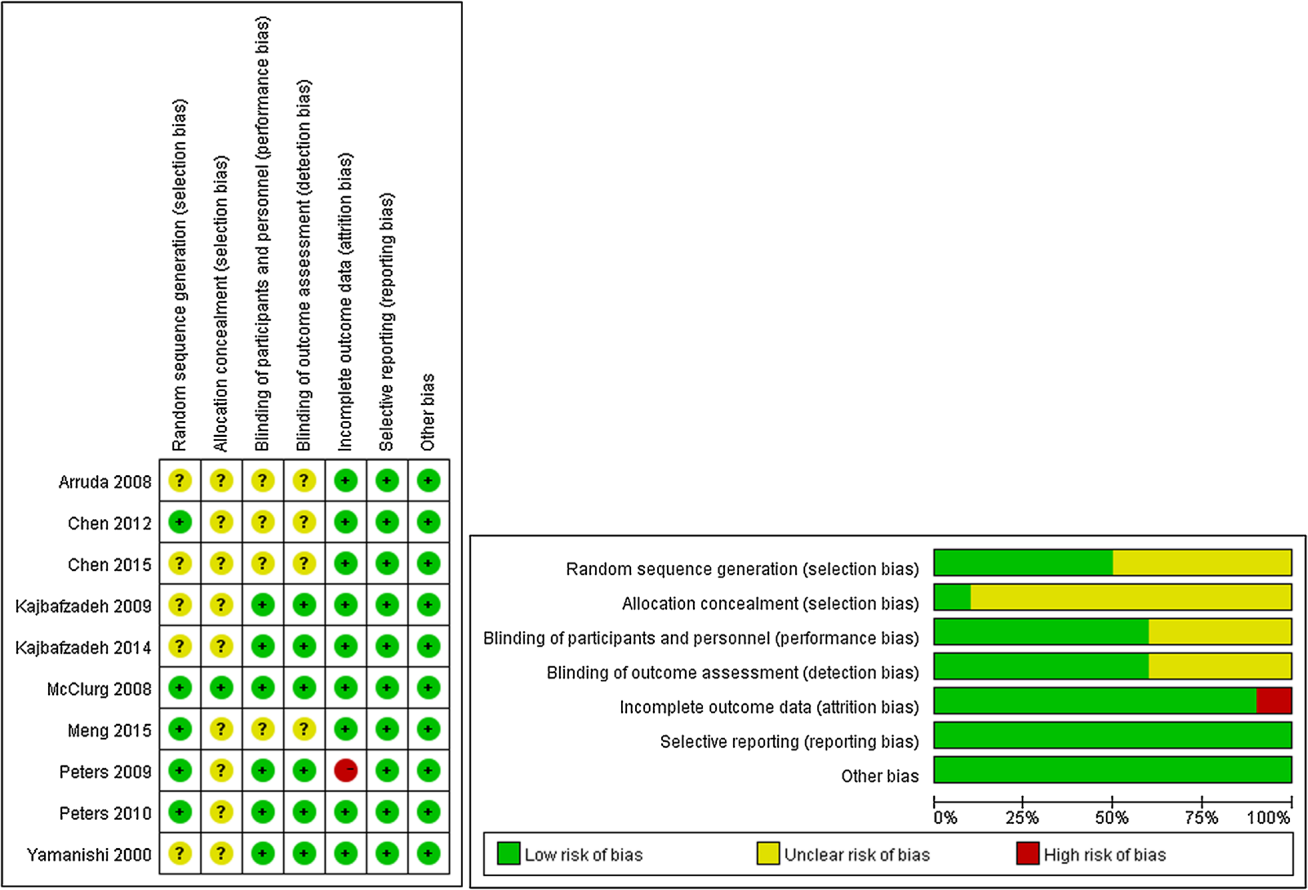
Assessment of the methodological quality of included studies

Results of assessment of methodological quality are displayed in Fig. 2.

## Meta-analysis results

### Maximum bladder capacity

**Fig. 3:**
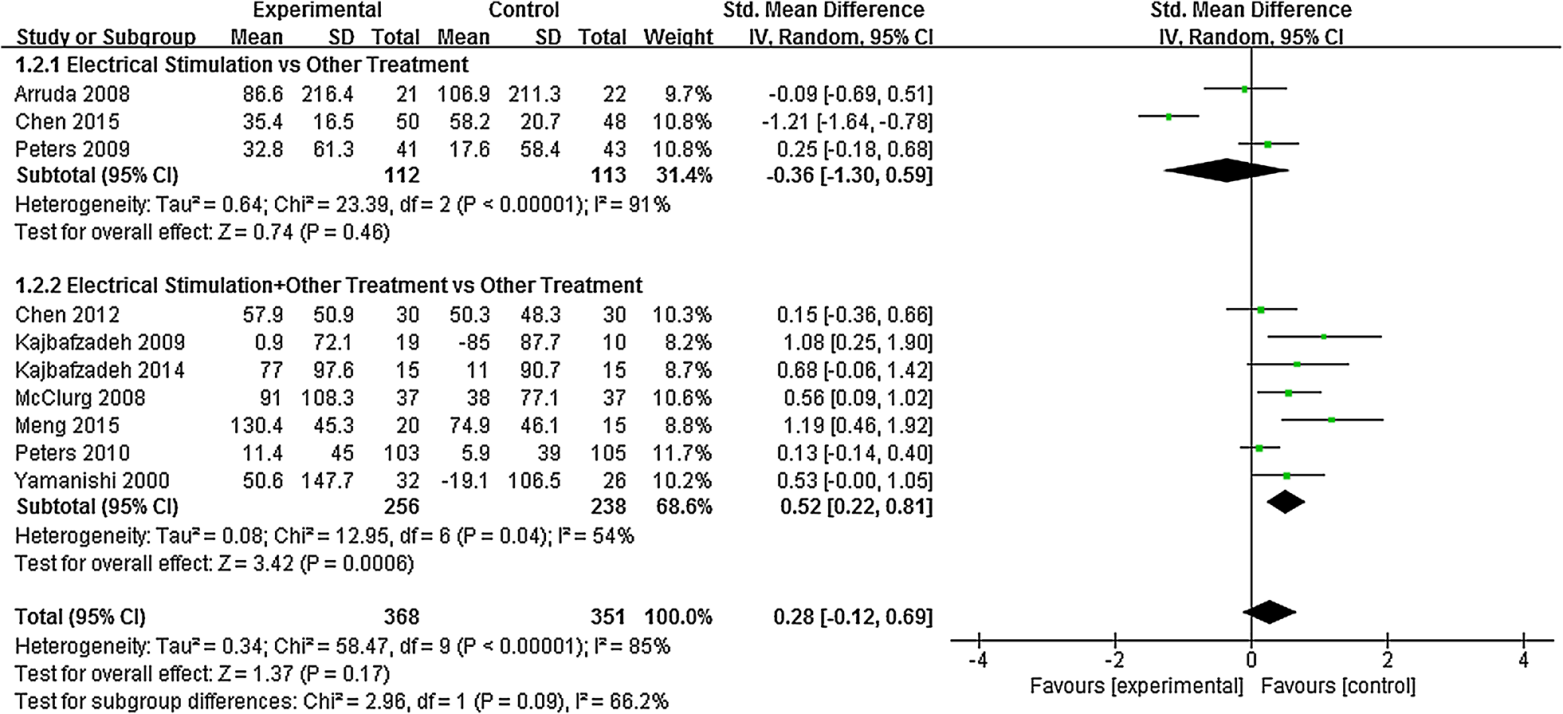
Meta-analysis of the maximum bladder capacity in patients with overactive bladder in both groups after treatment

For statistical heterogeneity among study results, subgroup analysis was conducted according to different interventions. For statistical homogeneity, random-effect models were used in the meta-analysis (Fig. 3). Results demonstrated significant differences in maximum bladder capacity between the two groups (ES + other therapies vs. other therapies) [SMD = 0.52, 95% CI (0.22, 0.81), *P* = 0.00006]. Mean bladder capacity of the treatment group increased by 45.6 mL, and the maximum increase was 130.4 mL. Mean bladder capacity of the control group increased by 14.6 mL, the maximum increase was 74.9 mL, and the maximum reduction was 85 mL. However, there was no significant difference in maximum bladder capacity in the ES group *vs*. other treatment group [SMD = −0.36, 95% CI (−1.30, 0.59), *P* = 0.46]. These findings suggested that ES combined with conventional therapy elicited better outcomes for increasing the maximum bladder capacity in OB patients than that in the control group. However, no significant difference was found between ES alone and other treatment alone.

### Maximum detrusor pressure

**Fig. 4:**
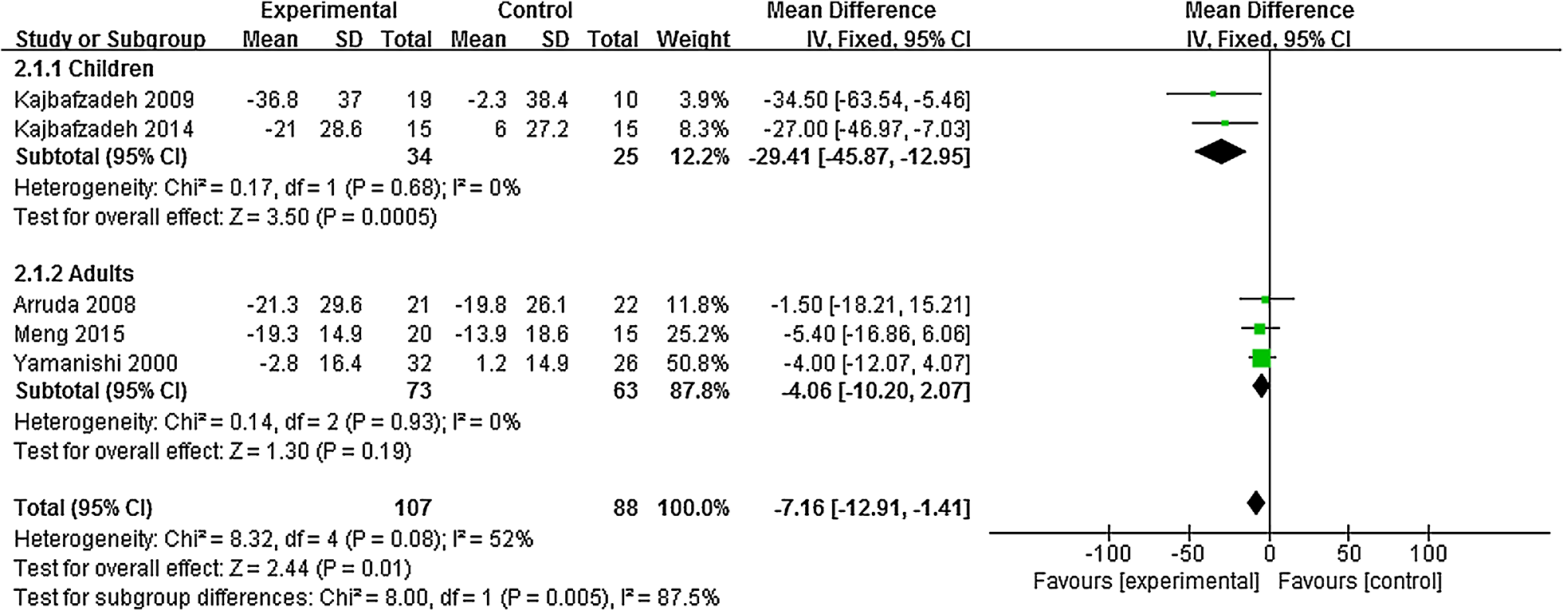
Meta-analysis of the maximum detrusor pressure in patients with overactive bladder in both groups after treatment

Five studies involving 195 patients were included for analyses of maximum detrusor pressure. For statistical heterogeneity among study results, subgroup analysis was conducted according to age. For statistical homogeneity, fixed-effect models were used in the meta-analysis (Fig. 4). Results showed significant differences in the maximum detrusor pressure between children and controls [MD = −29.41, 95% CI (−45.87, −12.95), *P* = 0.005], but no significant difference was identified between adults and controls [−4.06, (–10.20, 2.07), *P* = 0.19]. These results confirmed that ES elicited better effects for reducing the maximum detrusor pressure in children with OB than that in controls, but no significant difference was found between adults with OB and controls.

### Bladder compliance

**Fig. 5:**
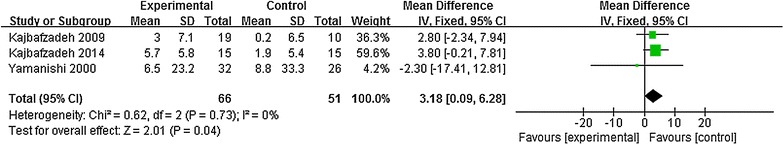
Meta-analysis of bladder compliance in patients with overactive bladder in both groups after treatment

Three studies involving 117 patients were included for analyses of bladder compliance. For statistical homogeneity, fixed-effect models were used in the meta-analysis (Fig. 5). Results showed significant differences in bladder compliance between the two groups [MD = 3.18, 95% CI (0.09, 6.28), *P* = 0.04]. Mean increase in the treatment group was 5.3 cmH_2_O/mL and in the control group was 5.0 cmH_2_O/mL. These data suggested that ES elicited better effects for improving bladder compliance in OB patients compared with the control group.

### Residual urine

**Fig. 6:**
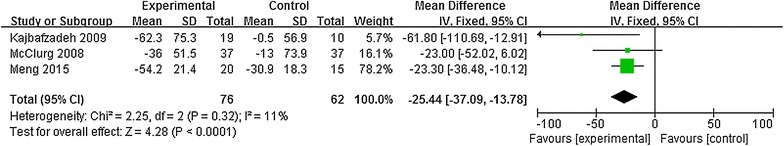
Meta-analysis of residual urine in patients with overactive bladder in both groups after treatment

Three studies involving 138 patients were included for analyses of residual urine. For statistical homogeneity, fixed-effect models were used in the meta-analysis (Fig. 6). Results demonstrated significant differences in residual urine between the two groups [MD = 25.44, 95% CI (13.78, 37.09), *P* < 0.0001]. In the treatment group, the mean reduction was 47.4 mL, and the maximum reduction was 62.3 mL. In the control group, the mean reduction was 15.3 mL, and the maximum reduction was 30.9 mL. These results suggested that ES elicited effects for reducing residual urine in OB patients compared with those in the control group.

### Frequency of enuresis

**Fig. 7:**
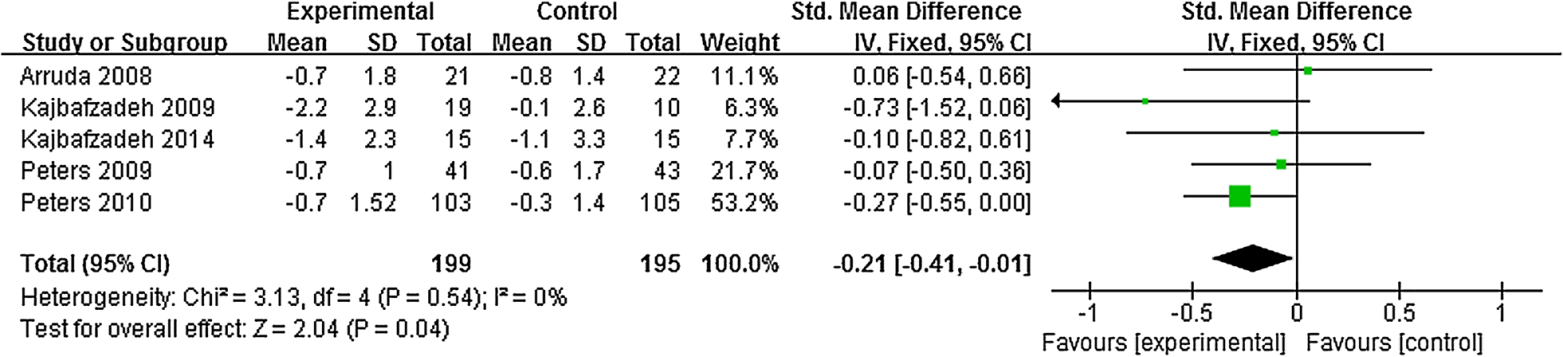
Meta-analysis of the frequency of enuresis in patients with overactive bladder in both groups after treatment

Five studies involving 394 patients were included for analyses of enuresis. For statistical homogeneity, fixed-effect models were used in the meta-analysis (Fig. 7). Results demonstrated significant differences in the frequency of enuresis between the two groups [SMD = –0.21, 95% CI (–0.41, –0.01), *P* = 0.04]. Mean reduction in the treatment group was 0.9 times, and in the control group was 0.5 times. These data suggested that ES elicited better effects for reducing the frequency of enuresis in OB patients than those in controls.

### Adverse reactions

Six studies reported adverse reactions to ES, which were fecal incontinence, discomfort, and muscle spasm at the site of stimulation (Chen et al. 2012). Adverse reactions to anticholinergic drugs included dry mouth, dry eyes and blurred vision (McClurg et al. 2008; Peters et al. 2009). Autonomic nerve reflexes and pyrexia appeared during injection of botulinum toxin type A, (Yamanishi et al. 2000) but severe adverse reactions did not occur. Three studies did not report obvious adverse reactions. The remaining study did not report adverse reactions.

## Discussion

Causes of OB are mainly neurogenic: injury to the spinal cord, multiple sclerosis, or spinal meningocele. Treatment of neurogenic bladder is very challenging. Some methods have been used (e.g., drugs, bladder rehabilitation training, surgery) but outcomes have been barely satisfactory. Moreover, long-term treatment is expensive, so many patients and their families cannot adhere to treatment. Thus, more effective treatment methods for OB have been studied.

In 1954, Boyce was the first to use ES: he employed ES in the bladder wall. In 1963, Caldwen undertook treatment of urinary incontinence. ES stimulates nerves and muscles with an electric current to initiate nerve reflexes and muscle contractions. In this way, the structure and function of the detrusor of the bladder, urethral sphincter and pelvic floor are altered (Craggs and McFarlane 1999). ES can be classified into stimulation at sacral nerves, pelvic floor, tibial nerve, or pudendal nerve.

The main functions of the lower urinary tract are urine storage and periodic urination, which are accomplished through the bladder and urethra, respectively. The lower urinary tract is controlled simultaneously by the sympathetic nerve (T10–L2), somatic nerve (S2–4), parasympathetic nerve (S2–4), the pontine micturition center, and the cerebral cortex. The bladder is also controlled by pelvic afferent nerves, which comprise mainly myelinated Aδ nerve fibers and some unmyelinated C nerve fibers. Somatic nerves mainly conduct sensations and pressure within the bladder. Coordination of sympathetic and somatic nerves controls bladder relaxation and sphincter contraction to achieve urine storage. Parasympathetic nerves primarily control detrusor, external urethral sphincter, and perineal muscles. The cerebral cortex sends messages on urine storage or micturition to the pontine micturition center. The latter controls the synergy between detrusor and urethral sphincters.

The main principle of ES is to activate peripheral sensory and motor nerves to produce sensations and muscle contractions, as well as to activate different spinal reflex zones or other central nervous systems, thereby resulting in the corresponding effect. Transmembrane currents are produced using an artificial external current to the outer membranes of neural cells or nerve fibers, thereby resulting in depolarization. If the current amplitude reaches a threshold value or less, it can stimulate action potentials, produce nervous excitation, and affect the particular organ. Therefore, urination by ES of nerve fibers is feasible. In accordance with the electrophysiological properties of the bladder and lower urinary tract, patients with bladder dysfunction have been subjected to ES. That is, during the micturition period, EC activates the motor nerve fibers that dominate the detrusor, cause bladder contraction, increase the pressure within the bladder, and promote bladder emptying. During the urine-storage period, EC suppresses the abnormal detrusor reflex, activates the muscles of the closed urethra, and prevents urine leakage (Jezernik et al. 2002).

Of the 10 RCTs included in our meta-analysis, five studies described the randomization method and procedure precisely,but only one study described allocation concealment. The remaining five studies mentioned only the method of randomization. Seven studies used a blinding method, including six double-blinded studies (mainly subject-blinded and evaluator-blinded). Reported data of one study were incomplete. All studies reported dropouts and the number of patients lost to follow-up. Only four studies reported the duration of follow-up. Most studies compared baseline data (e.g., patient age, proportion of males and females) and results demonstrated that baseline data were comparable between the trial group and control group. The methodological quality of most studies was high, so the results of this meta-analysis are a good reference for further studies.

The 10 RCT studies involving 719 patients mainly analyzed the efficacy and safety of ES for OB treatment. Our results showed that ES elicited better effects in OB patients than those in the control group because it increased bladder compliance and reduced residual urine and enuresis frequency. The effects of ES on reducing the maximum detrusor pressure were better in children than in controls, but a significant difference was not observed between adults and controls. The effects of ES combined with other therapies on increasing the maximum bladder capacity were better than for other therapies alone, but no significant difference was noted between ES alone and other therapies alone. In addition, we observed that, in male-dominated studies, ES mainly improved the bladder capacity whereas, in female-based studies, bladder compliance was also improved significantly in addition to bladder capacity. In children, ES improved the bladder capacity, mean maximum detrusor pressure, mean bladder compliance, and enuresis whereas, in adults, the main improvements were on the bladder capacity and enuresis.

Only one study of the 10 RCTs reported the adverse reactions to ES: fecal incontinence, discomfort and muscle spasm at the site of stimulation (Chen et al. 2012). However, adverse reactions may be different in different stimulation modes. Initially, the most widely used method is to stimulate sacral nerve roots. Such ES requires implantation of an electrical stimulator in the patient combined with resection of sacral nerve roots. The effect is obvious, but it can cause penile erection and ejaculation dysfunction, and this type of surgery is irreversible. Moreover, infection, pain, and failure of the electrode device can appear at the implant site (Bosch and Groen 2000; Kutzenberger 2007; Wang et al. 2007; Hino et al. 2010). Medical and engineering advances has led to minimally invasive (and even non-invasive surface) ES, (Lee et al. 2012b; Hagerty et al. 2007; Radziszewski et al. 2009; Radziszewski 2013; Gobbi et al. 2011) and ES can be used widely. Overall, the prevalence of adverse reactions of ES is low less: no deaths have been reported. Therefore, ES appears to be very safe.

Our meta-analysis demonstrated no significant differences between ES and other therapies with respect to the maximum bladder capacity and maximum detrusor pressure. However, these data do not mean that ES had no effect. Results of the 10 RCTs showed that ES was effective in improving bladder function and the symptoms of urinary incontinence, data that are in accordance with the outcomes of non-RCTs (Lombardi and Del Popolo 2009; Kabay et al. 2008, 2009). Studies have shown that the therapeutic effects of implantable sacral anterior root stimulation combined with sacral posterior root resection are remarkable, but such stimulation is invasive, requires considerable surgical skill, and is expensive. Such studies were not included in our meta-analysis because they were non-RCTs. According to the literature, the practical effects of ES in OB treatment should be better than that shown in our meta-analysis. Hence, to improve ES efficacy, scholars need to improve the methodology of further studies.

We found that the parameter settings of ES were different in different studies, as were the therapeutic effects. Irrespective of the type of ES used, there were no standard parameter settings, so the effects were limited. We consider treatment with ES to be in the exploratory stage. We cannot provide clear conclusions on the optimal form of ES and how to setup the relevant parameters of ES to maximize its therapeutic effect. Hence, ES cannot replace conventional therapy, but can be used as adjunctive therapy. We suggest that future studies should focus on six main areas. First, more refined ES will stimulate muscles and nerves accurately. Second, animal experiments can help to define the optimal parameters in various types of ES. Third, after identification of optimal ES, studies focusing on optimal ES and other therapies should be carried out to: maximize the therapeutic effect; shorten the treatment course; reduce the incidence of complications. Fourth, ES should be non-invasive. Fifth, close attention should be paid to improvement in the psychology, emotional state, and quality of life of patients. Finally, the effects of ES on improving the bladder function, physical activity and social inclusion of OB patients should be verified.

Our meta-analysis had seven main limitations. First, the number of RCTs and patients was small. Second, the included studies had been published. Third, we did not retrieve relevant “gray” literature. Fourth, RCTs with negative results may not have been published. Fifth, the duration of follow-up, illness, and treatment were not completely consistent in patients from different studies. Sixth, most studies did not describe allocation concealment. These six limitations could have resulted in bias in research findings. Seventh, because of the small number of RCTs, we compared and evaluated only ES and other therapies, and did not study the effects of different types and parameter settings of ES on outcomes. Taken together, the conclusions of our meta-analysis can be used only as a clinical reference.

## Conclusions

ES has certain effects on OBs, and severe adverse reactions have not been observed. Thus, ES is safe, efficacious, and worthy of clinical use. Limited by the quality and quantity of included RCTs, our conclusions should be confirmed by rigorously designed randomized, double-blind, controlled trials (especially large-sample, multicenter, randomized, double-blind, controlled trials).

## Abbreviations

OB: overactive bladder
ES: electrical stimulation
RCT: randomized controlled trial
MD: mean difference
SMD: standardized mean difference
CI: confidence interval
OR: odds ratio
